# A Retrospective and Prospective View of Dentistry

**Published:** 1861-02

**Authors:** W. A. Pease


DENTAL REGISTER OF THE WEST.

VOL. XV.] FEBRUARY, 1861. [NO. 2.

### Original Essays and Communications.

### A RETROSPECTIVE AND PROSPECTIVE VIEW OF DENTISTRY.

BY W. A. PEASE, M. D.

[Read in the Mad River Valley Dental Society.]

DURING the course of any progressive science or profession, it is useful to pause, at times, and see the progress it has made, and whither it is tending. This is the more useful and becoming in a young profession, that sprung suddenly into existence to supply a great public want, which, while it requires a high order of medical learning, and owes much of its prominence to the skill of its members in operating on the mouth and teeth, is also, as now practiced, based, to a considerable degree, on mechanical tact and ingenuity.

The profession of dentistry is anomalous. Though nearly allied to a specialty of the medical, yet, unlike it, it can not be truly said to have had any history, or any past,—it is a profession of the present, the offspring of civilization, and of the American climate and institutions.

It will fall to the province of this paper to notice, briefly, the causes in the history of our race, which prevented dentistry, as a profession, from coming sooner into existence as an auxiliary of the medical; and what were the peculiar circumstances, which, finally, gave it birth, a vigorous growth and development, unprecedented in the history of any other science or profession.

Man, as a natural animal, leads a life simple and bucolic. His wants are few, his tastes are easily gratified, and he is possessed of so healthy and robust a constitution, that, accidents excepted, he has little need for the services of a dentist. Without pausing to consider the autocthones of this country, whose dentures are admirable; if we extend our review to the Eastern Hemisphere, and to the ancient and modern civilizations, we will find that dentistry, in them, has never assumed the form and proportions of a profession; it has none of the attributes of a science, simply because there was no great public want to call it into existence.

Leaving modern Europe, for the present, if we trace back the history of our race to the ancient civilizations, we will find in their form of government, the structure of society, education, habits of life, and sustenance of the people, few elements likely to create a demand for dentists, and fewer to furnish the supply if the demand existed. Although, undoubtedly, there then existed some loss of teeth from caries as now; and from their warlike propensities, their primitive method of warfare, they were peculiarly liable to loss of teeth by violence; yet, it would fall, principally, upon that class of community, which would have little inclination, or ability to patronize a dentist. Neither had the ancient Greeks nor Romans any more need for dental interference than the Egyptians, or Syrians. Their habits of life, simple diet, abundance of out-door exercise, and the peculiar care they took to preserve their health and physical vigor, were eminently calculated to preserve their teeth. Dismissing, then, from our view the ancient civilizations, as having, from the very circumstances under which they existed, little inclination for or need of dentists, we will find, in the feudal times, the first germination of those circumstances and conditions of society, which, in after ages, were to call into existence the dental profession.

Shut up and isolated from society, in their old feudal castles, the haughty old barons were often forced, for considerable periods of time, to form a society of their own, out of the material with which they were surrounded. Here, woman first arose from the condition of a slave, or the minister to the pleasure of man, to be his companion, and to occupy a position more nearly akin to the one she has since held in modern Europe. Spreading from the old castles, as points of diffusion, the amelioration in the condition of woman extended from place to place, and from rank to rank, slowly it is true, but, steadily, until finally, after the lapse of centuries, it reached all nations, and nearly all classes. With her new conditions came new pleasures, duties, responsibilities and also new penalties. She acquired a greater delicacy of structure and sensibilty, together with a higher grade of education, which, if they did not so soon beget, made her sensible of annoyances, inconveniences, and ills to which she was before a stranger. Still actively participating in the pleasures of her lord, she was by no means a caged bird, pining for light and air, or a prey to lassitude. She frequently accompanied him to the chase, the tournament, or the camp; thus preserving all of the delicacy of the high, with much of the vigor, but, none of the coarseness of the servile condition. Thus, even to the present time, in the higher classes of Europe, the women, secure in and conscious of their position, in spite of the deteriorating influences of family intermarrying, by their active exercise, both in field and at home, have maintained a vigor of constitution, that has given them a perfection of denture, truly admirable. Born to a life of leisure and enjoyment, surrounded by servants to minister to every want, with houses filled with books, pictures, statuary—every thing that heart or taste can desire, they have found that all real, rational enjoyment—the true dignity of womanhood—consists in a life of activity, and, that the dignity of idleness is the dignity of ill health, and of self-dissatisfaction.

Turning then, from Europe, as presenting no great want to stimulate the ingenuity or the energies of man to supply the limited loss of natural teeth by artificial dentures, or to seek for remedial agents to cure, or prevent disease, we will find all of the conditions necessary to call forth the inventive genius of the mechanic to invent and perfect artificial dentures, and, of the physician and surgeon, to stay the rapid and general decay of the teeth, in America of the nineteenth century.

The class of people coming on to the stage of life thirty years ago were peculiarly situated. The country had enjoyed a season of repose and prosperity; the labors of the husbandman had yielded a bountiful return, which had enabled him to improve his farm, erect buildings, comfortable, commodious, and often elegant, to educate his children, and to surround the family with many of the elegancies and luxuries of life; while commerce, manufactures, and the professions had been equally prosperous. Remembering the privations he had endured, in the total absence of a hereditary aristocracy, or titled nobility, each head of a family strove to place his children in that position, in which they could profit the most by the circumstances by which they were surrounded, and attain to that high social and political position, due to merit and earnest endeavor. Thus, in a country where there was a pretty equal distribution of wealth, where society was founded on the natural laws of affinity, and the only aristocracy was that of merit, or the ability, or disposition to enjoy the luxuries and elegancies of life, there was a noble emulation and ambition to surround the family with those elegancies, which were then fresh in their minds, as belonging to, and which, they constantly associated with the aristocracy of Europe. Thus, the only external sign of an aristocracy in a democracy —the one which addressed itself to the age, and was the most cognizable, was a certain elegance in attire, in household surroundings and equipage. This desire, common to the people of this country, as soon as they have risen above the sphere of actual want, to conform to the requirements of taste in habiliments and equipage, paved the way for the rule of Fashion, and gave a certain force of law to whatever was done by any individual, who, by force of circumstances or taste, was recognized as an *arbiter eleganturium*, and leveled all up to his standard to restore his democratic balance. At this period in our history, there existed a large amount of dental disease in the mouths of our countrymen. Large classes of persons, especially females, past the meridian of life, had lost a considerable number of teeth; while many of them were toothless. This loss was severely felt; not only from the difficulty they experienced in masticating their food, in pronunciation, but, also, as a deformity, giving them a superannuated appearance. Here was a great, pressing, public want, common to all the cities and villages of the land; but, happily, a want, though difficult to supply, yet more easily supplied, than had it been equally pressing to save the natural teeth; because, this appealed directly to the mechanical ingenuity of a free and universally educated people; remarkably thoughtful, self-reliant, and given to invention,—to natural mechanics, fruitful in devices. Hence, this latent, unexpressed want had but to receive utterance; people had but to know that they could be supplied, however imperfectly, at their own homes, and for a reasonable compensation, instead of being obliged to send to Europe, as did Washington, and others, for a cumbersome, inefficient, and expensive piece of mechanism; then orders would be numerous, and a brisk trade would spring up. Especially would this be the case, if a set of teeth was inserted, for the right person, in the place; for then, independent of the intrinsic value it might be to him, it would have much of the force of fashion, and be adopted by the toothless accordingly. Distinctly is the excitement in social circles recollected, after a fashionable lady, in an interior town in New England, returned from

New York, with a new set of teeth. It was the first one ever brought to the place, and it excited a deal of gossip and curiosity; and although from its bad adaptation to the jaw, it caused her constant pain and trouble, it was worn most heroically, until, several months afterward, she suddenly sickened and died. Then it was asserted by the servants in the kitchen, and indeed, by others of a much higher order of intelligence, that she was removed by special interposition of Providence, for the sin of having dared to have restored, organs, of which she had been prematurely deprived, for some wise and inscrutable reason.

The artificial teeth used by Washington, and by others to a much later period, were carved to fit the mouth from solid slabs of ivory. Although, natural teeth were used, either pivoted on fangs, or mounted on some mechanical contrivance, that afforded a feeble support. They, in turn, were succeeded by porcelain teeth; and although superior to them in cleanliness, and durability, they were but a poor representative of them, either in form, color or expression; and it was not till near the time that Messrs. Jones, White & McCurdy commenced manufacturing them, that they were brought to such artistic perfection, as to make them very desirable substitutes for the natural teeth. After this time they were rapidly improved, in form, color, and expression; and improvement followed as rapidly on improvement, in the method of mounting them, till they were brought to very great perfection and usefulness.

Leaving the mechanical department, which we have seen took precedence of the operative, or dentistry proper, and from the tangibility of artificial teeth, and the real benefit they at length became to the wearer of them, and from the fact that they addressed themselves to the eye, and thus were noticed, and commented upon by others, they raised dentistry rapidly to a high position in the public estimation, as a useful and ornamental art, and paved the way for the advent of dentistry as a profession. Prior to 1820, Dr. L. S. Parmly had acquired a great reputation as a ntist, both in Europe and America; but his professional labors seem to have been chiefly directed to cleaning, regulating or extracting the teeth, or removing, by the file, superficial caries. At that time, deep-seated caries was beyond the resources of art, and hence, only those teeth having superficial cavities were ever plugged. The next step in the progress of conservative dentistry was to destroy the nerve, by means of a probe, or of arsenious acid, and then plug the cavity of decay; but this was soon found to produce so serious trouble, from acute and then chronic periostitis, that it was discontinued by many practitioners.

At the time the Baltimore College was established, dentists were to be found in all the centers of commerce or trade in the land; and it was then seen, from the premature and rapid decay of the teeth, that dentistry would soon present a wide field to men of mind and education, for lucrative and honorable employment, provided they could give it tone and direction, and raise it from a trade, requiring but a moderate amount of learning and skill, to the dignity of an independent profession, or a specialty of the medical. They hoped that men educated in a college, devoted specially to the profession, would soon be enabled to so far improve the practice as to make the mechanical department entirely subservient, and to occupy the same relative position to the dentist, that the manufacturer of artificial limbs or glass eyes does to the surgeon. From this time forward, during the decade ending in 1850, there was a gradual improvement in the art of plugging teeth, not only as to the quality of the plug, but also, as to the ability of plugging successfully a greater variety of the more difficult cavities; but it was not till 1854, when Dr. Ballard discovered that acute and chronic periostitis could generally be successfully treated, and thus, all the teeth in the mouth sufficiently strong to retain a plug, could be rendered valuable for years, if not for life, that dentistry could be said to preponderate over mechanical art, and thus to be established on a professional basis.

From this time there was no need, accidents excepted, that a person having moderately healthy teeth, who daily cleaned them, and had them plugged as soon as decay was apparent, should ever wear artificial teeth. Dentistry was now in the ascendant, and it was thought, as soon as the public mind could be disabused from the prejudice against plugs, caused by the miserable failures of mechanics and incompetent operators to make their plugs stick, or to protect the teeth, that the number of persons getting artificial teeth would be inconsiderable. Dentists now began to be hopeful, not only as to the future of their profession, but also as to the future of the race, for they saw nothing but disaster and degeneration, if mechanics were to go on and accelerate the loss of the teeth, by their ill-directed attempts at plugging them ; and afterwards, fill the mouths of many of the men and half of the women in the land with their artificial substitutes.

In dentistry, as in other arts, improvements come seldom alone. There are periods of rest or incubation, during which the inventive faculty seems dormant, and people are enjoying or testing the capabilities of the improvements they already possess. Suddenly we are startled by an improvement that is really valuable, and a decided step in advance ; and often, before the first exhilaration caused by the new discovery has subsided, come other, and perhaps still other improvements in the same, or other departments, that are independent of, and yet auxiliary to it, that give it a wider range of application and usefulness. Dr. Ballard had scarcely published his theory, that acute or chronic periostitis of the tooth was curable, than Mr. A. J. Watts produced a sponge gold, for plugging teeth, that rendered the discovery of Dr. Ballard of much greater importance ; and Prof. Arthur, experimenting on the capabilities of sponge gold, discovered that ordinary gold foil properly annealed, possessed many of the adhesive qualities of it ; and Dr. Clark also discovered that, from gold foil formed into cylinders, he could make superior plugs. These discoveries, coming one after another, placed operators in a very high position, and made the distinction between a dentist and a mechanic greater than it had ever been before; in short, in theory they destroyed the vocation of mechanical dentists, as a large and distinct class, as they ought to have done in practice. For their fear of periostitis being removed, with these materials, dentists were able to form and build up solid and durable plugs in large cavities, and restore the form, and strengthen frail and badly broken teeth, so that that large class of cases, which neglect, or previous maltreatment, had rendered nearly hopeless, were now generally manageable. Convinced themselves of the intrinsic value of these improvements, and of the command they gave them over disease, dentists imagined that they had but to show to the community the superiority of the new practice, to have it as gladly adopted as it had been by the profession; and that as their practice had been revolutionized, there would soon be as great a revolution in that of the people, and that the demand for mechanical dentistry would soon visibly decrease. In this they were mistaken; they did not calculate upon the *vis inertia* to be overcome in all public improvement, upon the difficulty of changing the current of men's minds, or the practice of their lives; of convincing them that the new, more tedious, laborious and expensive method of plugging teeth possessed a greater intrinsic value than the old; that having often been deceived, they would view this as another of the new and great improvements constantly being made and heralded abroad by dentists of the sensation school, to be quickly abandoned and superseded by others. They did not see the relative position they occupied to the public clearly and distinctly, or whither they were drifting; that, whereas the physician could prescribe and administer his own prescription, the practice of the dentist being chiefly operative, he was obliged to consult the wishes of his patient, who, if his prejudices or whims were not complied with, could go to others more accommodating and pliable; besides, he could fix his own terms, having no fear of death before him; or seldom impelled by pain, he could deliberate, and go from one to another without greater risk than the loss of the tooth. Thus there is a depressing element in the dental profession, that weighs it down towards the level of a trade, that is not to be found in the medical, where acute sickness opens the purse strings, or in the legal, where titles, passions, or large money considerations prompt to the employment of the best counsel. This depressing effect is visible in practice; for the fees of the generality of practitioners are a low minimum, bringing them into constant conflict with their sense of duty to their patient, on one side, and the temptation, by imperfect operations, to secure for themselves a small profit on the other.

Few dentists have the moral heroism to so establish their fee bill as to secure for themselves a fair compensation, and for their patients the best of operations; they can not consent to limit their practice, to see people leave their office, day after day and year after year, because they require a fee that will enable them to deal justly and honorably with their patients, and at the same time, by study and the purchase of more and better instruments, prepare themselves for a wider field of usefulness; while their neighbors, by means of smaller fees and much poorer operations, secure an equal profit, a liberal patronage, and a popular reputation. Thus the whole course of dental practice,—the inevitable logic of events, points, unerringly, to one of two positions that must be occupied by dentists at no distant day—they must either be members of a profession or a trade; they can not be both. If they choose the former, they must seek, by means of high qualifications and the best of operations, to show the community that it is needless, that it is folly, to lose their teeth and to wear artificial ones; on the contrary, if they choose the latter, all they have to do, to secure the greatest success, is to perform their cheap, but valueless operations, ruin the teeth, under the guise of saving them; and thus, thoroughly convince the public that all operations are humbugs, and mechanics alone are worthy of patronage. Already, in all the principal cities, there are numerous persons engaged in the business, who manufacture artificial dentures that, so far as the public can judge, possess all of the requisites for comfort and usefulness, and that, too, at a price so little above the actual cost of production as to leave them scarcely mechanics' wages. Without the greatest exertion, this class of persons is going to increase, and vitiate still further the public sentiment; for every set of teeth that is inserted is an advertisement, it is known to and criticized by a large circle of acquaintances. The remedy for this will consist in making each filling as perfect as possible, and in convincing the public that the teeth should be plugged when the cavities are small. Where no teeth are lost, of course, none will be inserted.

